# Survival of mature T cells in the periphery is intrinsically dependent on GIMAP1 in mice

**DOI:** 10.1002/eji.201646599

**Published:** 2016-11-25

**Authors:** Preeta Datta, Louise M.C. Webb, Inxhina Avdo, John Pascall, Geoffrey W. Butcher

**Affiliations:** ^1^ Laboratory of Lymphocyte Signalling and DevelopmentThe Babraham InstituteCambridgeUnited Kingdom

**Keywords:** Apoptosis, CD4^+^ T cells, CD8^+^ T cells, Cell survival, Homeostasis

## Abstract

An effective immune system depends upon the survival of mature T cells in the periphery. Members of the GIMAP family of GTPases have been proposed to regulate this homeostasis, supported by the paucity of peripheral T cells in rodents deficient for either GIMAP1 or GIMAP5. It is unclear whether this lack of T cells is a consequence of an ontological defect, causing the thymus to generate and export T cells incapable of surviving in the periphery, or whether (alternatively or additionally) mature T cells intrinsically require GIMAP1 for survival. Using the *ER^T2^Cre^+^* transgene, we conditionally deleted *Gimap1* in C57BL/6 mice and demonstrate that GIMAP1 is intrinsically required for the survival of mature T cells in the periphery. We show that, in contrast to GIMAP5, this requirement is independent of the T‐cells' activation status. We investigated the nature of the survival defect in GIMAP1‐deficient CD4^+^ T cells and show that the death occurring after GIMAP1 ablation is accompanied by mitochondrial depolarization and activation of the extrinsic apoptotic pathway. This study shows that GIMAP1 is critical for maintaining the peripheral T‐cell pool in mice and offers a potent target for the treatment of T‐cell‐mediated diseases.

## Introduction

The functioning of the adaptive immune system is dependent upon the availability of a large pool of quiescent lymphocytes with diverse Ag receptors. These cells are continuously produced by the thymus from where they egress into the periphery as naive T cells with lifespans ranging from 3 to 10 months in mice 1, 2 to an estimated half‐life of 2 years in humans^3^. Long‐term survival and homeostasis of peripheral naive T lymphocytes is regulated by the availability of the γ_c_‐chain cytokine IL‐7 and of low affinity self‐peptides 4, 5.

The GIMAPs (GTPases of the IMmunityAssociated Proteins) are a family of 7–9 small GTPases highly expressed in lymphocytes 6. They are defined by the presence of an AIG1 domain, the prototype for which is found in *Arabidopsis thaliana*, where it is thought to play a role in defence against pathogens 7. The GIMAP family plays an important but as yet undefined role that contributes to the survival of cells during T‐ and B‐cell lymphopoiesis. Loss of individual members of the family has a detrimental effect on mature lymphocyte survival. For instance, significant T‐cell lymphopenia is observed in GIMAP5‐deficient mice and rats 8, 9, 10, 11. Moreover, the GIMAP5 lesion is also found to compromise the lifespan of rat peripheral T cells in vitro by increasing the rate of apoptosis 12. B cell lymphopenia is also observed in GIMAP5‐deficient mice 9, 13. Knock‐down of mouse *Gimap*3 in fetal thymic organ culture (FTOC) leads to a block in positive selection during thymopoiesis, while reconstitution experiments using GIMAP3‐deficient bone marrow also demonstrate defective T‐cell development and loss of the mature T‐cell populations 14. The double ablation of *Gimap3* and *Gimap5* produces a more severe phenotype than either individual deficiency 10. Preliminary analysis of GIMAP6‐deficient mice also shows its requirement for normal T‐cell survival in the periphery (John Pascall, personal communication). By contrast, mice and rats deficient in either GIMAP4 or GIMAP8 show no obvious defects in T or B cell lymphopoiesis 15, 16, 17 but ex vivo their T cells demonstrate delays in progression through apoptosis 15, 16.

GIMAP1 is the prototypic member of the family 18 and has a significant effect on lymphocyte survival. The gene is upregulated in response to p53‐mediated apoptosis in a temperature‐sensitive leukemia cell line 19; it is also reported to be upregulated in response to TCR stimulation under T_H_1‐polarising conditions and correspondingly down‐regulated under T_H_2‐polarising conditions 20.

The GIMAP1 protein is consistently expressed at all stages of thymopoiesis and its expression is maintained at a high level in mature lymphocytes 22. It is also expressed in non‐lymphoid tissues such as the brain, heart, lungs, and kidneys 21. To circumvent any issues of animal viability, we generated the first conditional mouse knock‐out model for the GIMAPs, by the hCD2iCre‐driven ablation of *Gimap1* in lymphoid progenitors 22. The early stages of lymphocyte development in the resulting mice appeared largely unaffected by this gene ablation. However, the mature T‐ and B‐cell compartments exhibited profound lymphopenia^22^. It was unclear whether the T‐cell deficit observed in these animals was the consequence of a late‐stage intrathymic defect that produced T cells incapable of surviving in the periphery (a “legacy effect”), or whether mature CD4^+^ and CD8^+^ T cells themselves depend intrinsically on GIMAP1 for their long‐term survival. The hCD2iCre‐conditional ablation model was unable to resolve this issue, not least because of the extreme paucity of mature cells remaining in the system. In addition, CD4 and CD8 SP thymocytes from *Gimap1^f/f^hCD2iCre^+^* mice were reduced in number and showed a survival defect in vitro. This suggested that the survival defect may occur before cells enter the periphery, implicating a “legacy effect” for peripheral T cells that have developed in the absence of GIMAP1 expression. To address this issue we have now generated an inducible *Gimap1* ablation model, based on the ER^T2^Cre system, in which a ‘floxed’ target gene may be electively ablated by the application of tamoxifen, or its derivative 4‐hydroxytamoxifen (4‐OHT), to otherwise normal cells. This allows selective ablation of GIMAP1 in mature T cells enabling us to determine if GIMAP1 is intrinsically required for their survival in the periphery.

In the present study we show that loss of GIMAP1 significantly compromises the survival of ex vivo‐cultured mature CD4^+^ T lymphocytes and of CD4^+^ and CD8^+^ T cells in vivo. We show that GIMAP1 is essential for the survival of both resting and activated CD4^+^ T cells. Closer examination revealed that the cell death observed in GIMAP1‐deficient CD4^+^ T cells was preceded by loss of mitochondrial function and activation of the extrinsic apoptotic pathway.

## Results

### Resting and activated peripheral CD4^+^ T cells require GIMAP1 for their survival ex vivo

**Figure 1:**
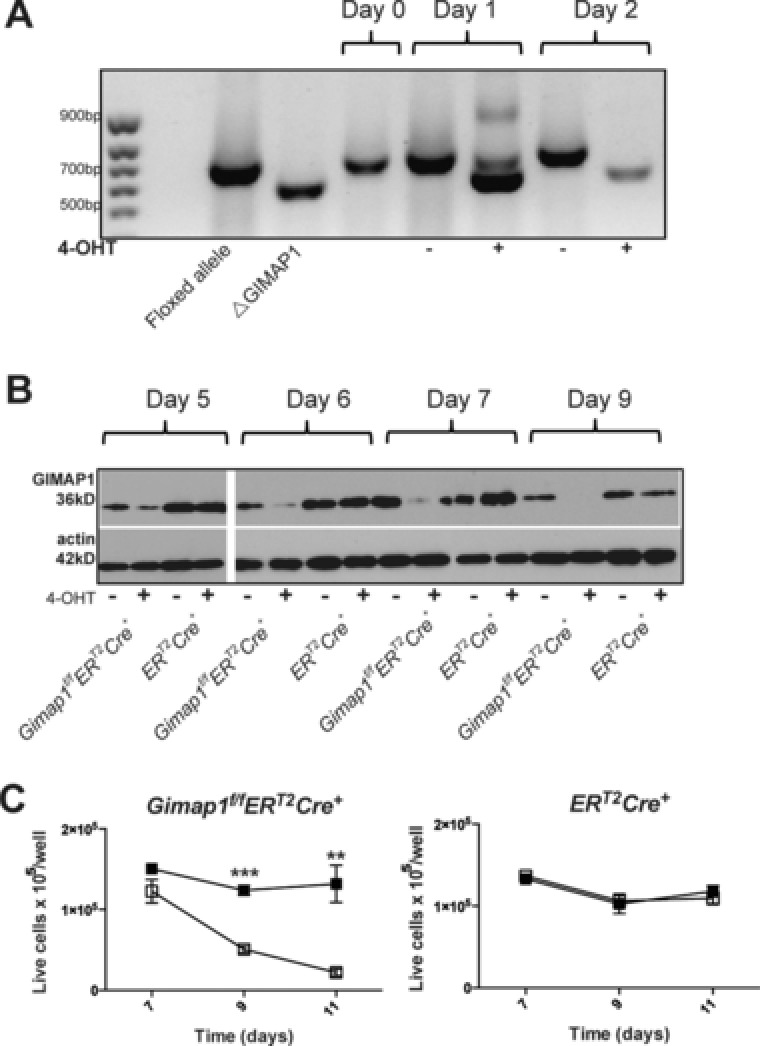
Conditional deletion of the “floxed” *Gimap1* allele by the *ER^T2^Cre* transgene and 4‐OHT treatment. (A) Deletion of floxed *Gimap1* gene. Resting CD4^+^ T cells from *Gimap1^f/f^ER^T2^Cre^+^ and ER^T2^Cre^+^* mice were cultured in IL‐7 in the presence of 50 nM 4‐OHT or vehicle control. Cells were harvested and DNA extracted prior to PCR analysis of *Gimap1* to determine deletion. (B) Western blot analysis of GIMAP1 and actin expression in CD4^+^ T cells from *Gimap1^f/f^ER^T2^Cre^+^* or *ER^T2^Cre^+^* mice cultured in IL‐7 in the presence of 4‐OHT or vehicle control. Actin was used as a loading control. (A, B) Data are from a single experiment representative of three experiments performed. (C) Resting CD4^+^ T cells were cultured in IL‐7 at a density of 1 × 10^5^/well in 96‐well plates. Cells were treated for two days with either vehicle control (DMSO: ■) or 50 nM tamoxifen (□). Live cells (DAPI^−^) were enumerated by flow cytometry. Results are presented as number of live cells/well and shown as mean ± SEM of triplicate samples. Data are from a single experiment representative of three experiments performed. ^**^ *p* < 0.005, ^***^ *p* < 0.0005 (paired 2‐tailed Student's *t*‐test).

Previously, we showed that deletion of *Gimap1* in early lymphoid progenitors resulted in a profound deficiency in peripheral T and B lymphocytes 22. It remained unresolved, however, whether the lack of T cells in *Gimap1^f/f^CD2Cre^+^* animals is due to (A) a requirement for GIMAP1 during development in primary lymphoid organs which impacts on their subsequent ability to survive in the periphery (a “legacy effect”), or (B) a requirement for continuous expression of GIMAP1 in peripheral T cells. To distinguish between these two possibilities, we developed a model in which *Gimap1* could be deleted in T cells once they had matured and entered the periphery. *Gimap1^f/f^* and *ER^T2^Cre^+^* mice were crossed to generate *Gimap1^f/f^ER^T2^Cre^+^* mice, with which we could perform conditional ablation of *Gimap1*with either tamoxifen or its derivative, 4‐OHT. To control for off‐target effects and Cre‐mediated toxicity, all experiments included cells from the *ER^T2^Cre^+^* mouse strain as a control 23. We first asked whether resting CD4^+^ T cells required GIMAP1. CD4^+^ T cells were purified from *Gimap1^f/f^ER^T2^Cre^+^* and *ER^T2^Cre^+^* spleen and lymph nodes and cultured in vitro in IL‐7 for 7–10 days in the presence of either vehicle (DMSO) or 4‐OHT. DNA was purified from cells to assess deletion of *Gimap1* and cell lysates were analysed for GIMAP1 protein expression by western blot. As shown in Fig. 1A, *Gimap1* was deleted by day 2 of culture. However, western blots showed that GIMAP1 protein was detectable until day 6–7 of culture and was completely lost by day 9 (Fig. 1B). To determine how deletion of *Gimap1* affects survival of CD4^+^ T cells, live cells were enumerated by flow cytometric analysis of DAPI^−^ cells using flow count beads. 4‐OHT had no effect on cell viability until days 7–9 of culture when the number of live *Gimap1^f/f^ER^T2^Cre^+^* CD4^+^ T cells plummeted (Fig. 1C). This correlated with the disappearance of GIMAP1 protein, suggesting that CD4^+^ T cells require continuous expression of GIMAP1 for their survival. No effect of 4‐OHT on the survival of *ER^T2^Cre^+^* T cells was seen (Fig. 1C), indicating that the effects on *Gimap1^f/f^ER^T2^Cre^+^* cells were not due to Cre‐mediated toxicity.

**Figure 2:**
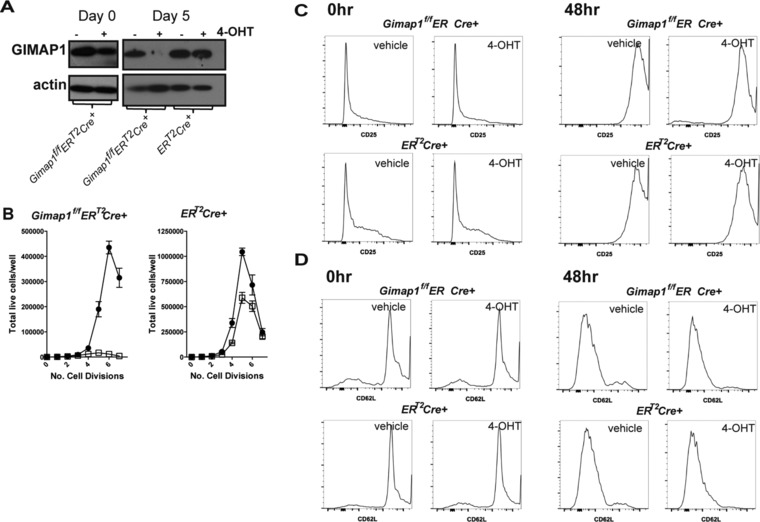
Activated CD4^+^ T cells require GIMAP1 for proliferation. CD4^+^ T cells were cultured in IL‐7 ± 4‐OHT for 5 days prior to CFSE staining and activation. (A) Expression of GIMAP1 on days 0 and 5 of activation was determined by western blot. Actin served as a loading control. Data are from a single experiment representative of three experiments performed. (B) Enumeration of live cells and the number of divisions undergone by day 5 post activation for *Gimap1^f/f^ER^T2^Cre^+^* and *ER^T2^Cre^+^* CD4^+^ T cells were pre‐treated with either vehicle control (●) or 4‐OHT (□) prior to staining with CFSE and activation with anti‐CD3 + anti‐CD28 in the presence of IL‐2. Dye dilution was evaluated by flow cytometry and shown as mean + SEM of three samples/replicates from one experiment representative of three experiments. (C) CD25 expression during activation. *Gimap1^f/f^ER^T2^Cre^+^* and *ER^T2^Cre^+^*CD4^+^ T cells from vehicle‐ and 4‐OHT‐treated cultures were stained with anti‐CD25 on day 0 and day 2 of activation and measured by flow cytometry. (D) CD62L expression during activation. *Gimap1^f/f^ER^T2^Cre^+^* and *ER^T2^Cre^+^*CD4^+^ T cells from vehicle‐ and 4‐OHT‐treated cultures were stained with anti‐CD62L on day 0 and day 2 of activation. (C, D) Data shown are from a single experiment representative of three experiments.

Published work on GIMAP5 has suggested that it participates in T‐cell activation 12, 24, 25 and that cell death in GIMAP5‐deficient lymphocytes can be overcome by activating the cells. However, the model systems used relied upon T cells that had developed in GIMAP5‐deficient rodents, making it difficult to determine whether the effects observed were due to a “legacy” effect. Furthermore, cells developing in a lymphopenic environment undergo homeostatic proliferation and acquire a “memory” phenotype, thereby making them a poor comparison for normal WT peripheral T cells. By using cells from *Gimap1^f/f^ER^T2^Cre^+^* animals, we were specifically able to address the role of GIMAP1 at a point after normal T‐cell maturation. We chose to ablate GIMAP1 during the IL‐2‐driven expansion phase following T‐cell receptor (TCR) stimulation, a stage where cells are undergoing rapid proliferation. *Gimap1^f/f^ER^T2^Cre^+^* and *ER^T2^Cre^+^*CD4^+^ T cells were first cultured for 5 days in IL‐7 in the presence of either 4‐OHT or vehicle and were then activated via CD3 and CD28 in the presence of exogenous IL‐2. This design enabled T cells to be activated whilst they still expressed GIMAP1 since GIMAP1 protein was detectable at the point of activation but absent by day 5 of activation (Fig. 2A). Cells were stained with carboxyfluoresceindiacetatesuccinimidyl ester (CFSE) immediately prior to activation to allow assessment of the number of divisions that each cell underwent. Live cells were enumerated by DAPI staining and Flow count beads during flow cytometric analysis. GIMAP1‐deficient CD4^+^ T cells failed to undergo the expansion phase characteristic of T‐cell activation (Fig. 2B) despite normal T‐cell activation (as determined by induction of CD25 expression and down‐modulation of CD62L; Fig. 2C and D). Very few live *Gimap1^f/f^ER^T2^Cre^+^* cells were detected by day 5 post‐activation, in comparison to control *ER^T2^Cre^+^* cells.

### The extrinsic apoptotic pathway is engaged in GIMAP1‐deficient CD4^+^ T cells

**Figure 3:**
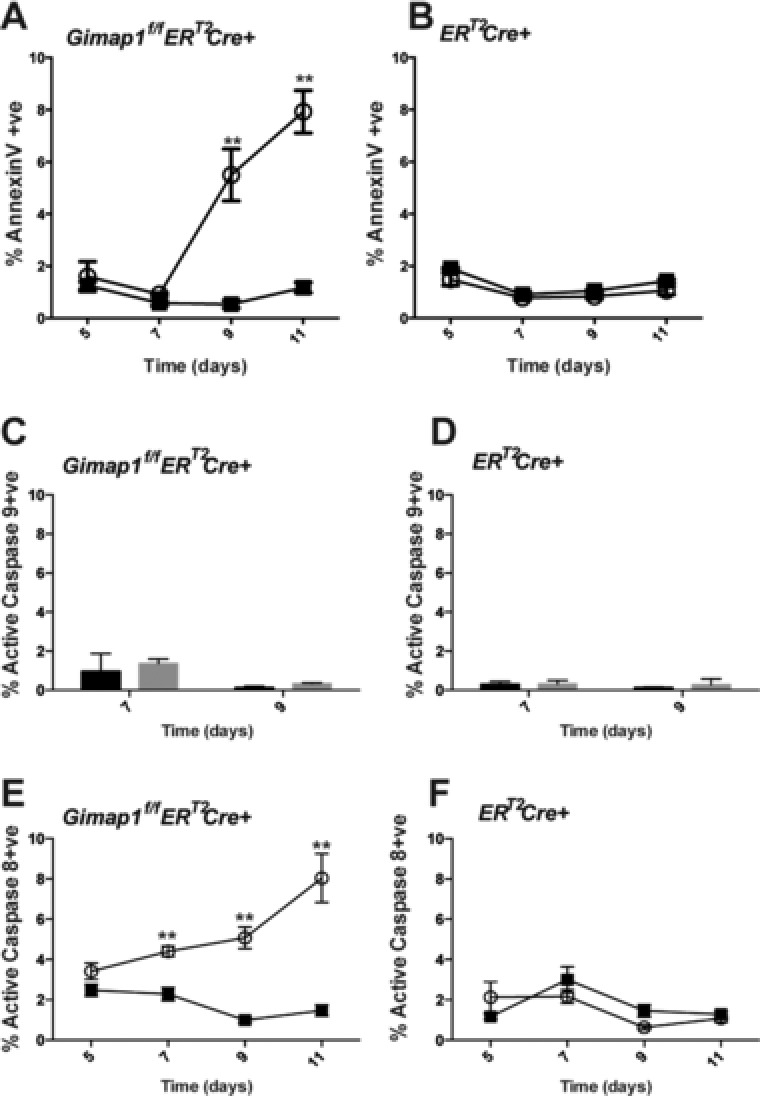
Extrinsic apoptosis in GIMAP1‐deficient CD4^+^ T cells. (A, B) CD4^+^ T cells from (A) *Gimap1^f/f^ER^T2^Cre^+^*or (B) *ER^T2^Cre^+^* mice were cultured in IL‐7 in the presence of vehicle control (■) or 4‐OHT (❍). The percentage of annexin V^+^cells within the DAPI^−^ population of CD4^+^T cells was measured by flow cytometry. (C, D) CD4^+^ T cells expressing activated caspase‐9 from (C) *Gimap1^f/f^ER^T2^Cre^+^* or (D) *ER^T2^Cre^+^* mice treated with vehicle (black bars) or 4‐OHT (grey bars). (E, F) CD4^+^T cells from (E) *Gimap1^f/f^ER^T2^Cre^+^*or (F) *ER^T2^Cre^+^* mice were cultured in IL‐7 in the presence of vehicle control (■) or 4‐OHT (❍). The percentage of caspase‐8^+^cells within the DAPI^−^ population of CD4^+^ T cells was measured by flow cytometry. Results are shown as mean ± S.E.M. for triplicate samples from a single experiment representative of three experiments performed. ^**^ *p* < 0.005 (paired 2‐tailed Student's *t*‐test).

The function of GIMAPs within lymphocytes remains obscure and the mechanism by which they act remains elusive. By using the *ER^T2^Cre^+^* transgene, we were able to look at the earliest events following *Gimap1* ablation, prior to ultimate cell death. We used our in vitro culture system to investigate which cell death pathways were engaged in GIMAP1‐deficient CD4^+^ T cells. First, we determined whether cells were dying via apoptosis by determining the percentage of annexin V^+^DAPI^−^(early apoptotic) cells and the kinetics of their appearance. As shown in Fig. 3A and B, apoptotic cells appeared on day 9 of culture and were only seen in 4‐OHT treated *Gimap1^f/f^ER^T2^Cre^+^* T‐cell cultures. Apoptosis is the result of a cascade of signals emanating from the initiator caspases, caspase‐8 and caspase‐9, which are themselves activated by distinct stimuli 26, 27. Caspase‐9 is activated during the intrinsic apoptotic pathway (usually the result of changes in Bcl‐2 family member expression), while caspase‐8 is involved in the extrinsic apoptosis pathway (activated in response to cell surface death‐receptor ligation) 27. We found no increase in the number of cells expressing activated caspase‐9 when GIMAP1 was deleted (Fig. 3C and D). We also found no differences in the levels of Bcl‐2 family members (Bcl‐2, Bcl‐x_L_, Mcl‐1, Bim, and Bax) in GIMAP1‐deficient cells at early time points (Supporting Information Fig. 1). We did find a significant increase in the percentage of cells expressing activated caspase‐8 in GIMAP1‐deficient cells (Fig. 3E and F). This was evident on day 7 of culture, i.e. prior to phosphatidylserine exposure (measured by annexin V binding), implicating caspase‐8 as the initiator of apoptosis in GIMAP1‐deficient CD4^+^ T cells.

**Figure 4:**
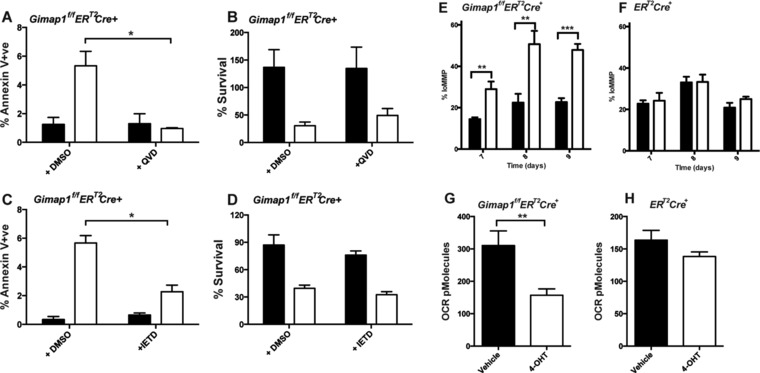
Caspase‐3 and caspase‐8 inhibition prevent apoptosis induction in GIMAP1‐deficient CD4^+^ T cells. (A, B) CD4^+^ T cells from *Gimap1^f/f^ER^T2^Cre^+^* mice were cultured in IL‐7 in the presence of vehicle control (filled bars) or 4‐OHT (open bars) plus either DMSO or QVD. (A) The percentage of annexin V^+^cells within the DAPI^−^ population of CD4^+^ T cells was measured by flow cytometry. (B) Percentage survival on day 9 of culture (relative to day 5 of culture) was determined by enumerating the number of DAPI‐ve cells. (C, D) CD4^+^ T cells from *Gimap1^f/f^ER^T2^Cre^+^* mice were cultured in IL‐7 in the presence of vehicle control (filled bars) or 4‐OHT (open bars) plus either DMSO or IETD. (C) The percentage of annexin V^+^cells within the DAPI^−^ population of CD4^+^ T cells was measured by flow cytometry. (D) Percentage survival on day 9 of culture (relative to day 5 of culture) was determined by enumerating the number of DAPI‐ve cells. (E, F) CD4^+^ T cells from (E) *Gimap1^f/f^ER^T2^Cre^+^*or (F) *ER^T2^Cre^+^* mice were cultured in IL‐7 in the presence of vehicle control (filled bars) or 4‐OHT (open bars). The percentage of cells with lo MMP within the DAPI^−^ population of CD4^+^ T cells was measured by flow cytometry. (G, H) CD4^+^ T cells from (G) *Gimap1^f/f^ER^T2^Cre^+^*or (H) *ER^T2^Cre^+^* mice were cultured in IL‐7 in the presence of vehicle control (filled bars) or 4‐OHT (open bars) for 7 days prior to determining oxygen consumption rate with a Seahorse analyzer. Results are shown as mean ± SEM for triplicate samples from a single experiment representative of three experiments performed. *p < 0.05, **p < 0.005, ***p < 0.0005 (paired 2‐tailed Student's *t*‐test).

To verify that caspase‐8 was involved in apoptosis of GIMAP1‐deficient CD4^+^ T cells, we looked at the effect of IETD, a caspase‐8‐specific inhibitor, on annexin V binding and cell survival of 4‐OHT‐treated *Gimap1^f/f^ER^T2^Cre^+^* cells. As shown in Fig. 4A & C, both IETD and QVD (a broad caspase inhibitor) inhibited apoptosis in 4‐OHT‐treated *Gimap1^f/f^ER^T2^Cre^+^* T cells (as determined by annexin V binding on day 9 of culture). However, neither inhibitor reversed the cell death (defined by lack of DAPI staining and cell enumeration on day 9 of culture) seen in 4‐OHT‐treated *Gimap1^f/f^ER^T2^Cre^+^* T cells (Fig. 4B and D). This suggested that the mechanism of cell death was either independent and/or upstream of caspase‐8 activation. We went on to investigate the effect that GIMAP1 deletion has on mitochondrial membrane potential (MMP) using the dye JC‐1 which exhibits mitochondrial potential‐dependent fluorescence and is widely used to monitor mitochondrial health 28. As shown in Fig. 4E & F, there was an increase in the percentage of GIMAP1‐deficient CD4^+^ T cells with damaged mitochondria (low MMP) by day 7. This was verified by measuring the oxygen consumption rate (OCR) in these cells on day 7 of culture (Fig. 4G and H).

### Activation of the extrinsic apoptotic pathway in GIMAP1‐deficient CD4^+^and CD8^+^ T cells in vivo

**Figure 5:**
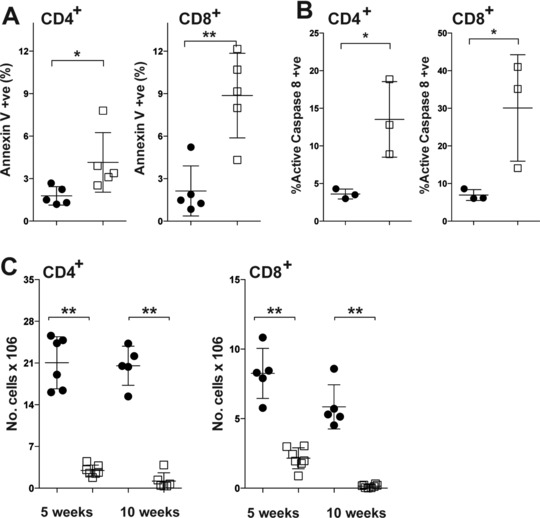
T cells from GIMAP1‐deficient mice are undergoing extrinsic apoptosis. (A) CD4^+^and CD8^+^splenocytes from *Gimap1^f/f^CD2Cre^+^* (□) and control *Gimap1^f/f^*(●) mice were stained with annexin V and DAPI. The percentages of annexin V^+^ cells within the DAPI^−^ population for individual mice were evaluated by flow cytometry and are shown as mean ± S.D. and are from a single experiment representative of three experiments performed. (B) CD4^+^and CD8^+^splenocytes from *Gimap1^f/f^CD2Cre^+^* (□) and control *Gimap1^f/f^*(●) mice were stained for activated caspase‐8 and DAPI. The percentage of cells expressing activated caspase‐8 within the DAPI^−^ population for individual mice is shown as mean ± SD and is from a single experiment representative of three experiments performed. (C) CD4^+^splenocytes from 5 and 10 week‐old *Gimap1^f/f^CD2Cre^+^* and *Gimap1^f/f^* mice were determined by flow cytometry. Results are presented as numbers of CD4^+^(left) and CD8^+^ (right) T cells per spleen for *Gimap1^f/f^ER^T2^Cre^+^* (□) and control *Gimap1^f/f^* (●) mice and shown as the mean ± S.D. Data are from a single experiment representative of three experiments performed. ^*^ *p* < 0.05, ^**^ *p* < 0.01 (unpaired 2‐tailed Student's *t*‐test).

We went on to verify our in vitro findings by looking at levels of active caspase‐8 expression and annexin V binding in naïve CD4^+^and CD8^+^ T cells *Gimap1^f/f^CD2Cre^+^* mice. Peripheral T cells from these animals displayed an increased proportion of early apoptotic cells (annexin V^+^DAPI^−^) and cells expressing active casapse 8 (Fig. 5A and B). Thus, it appears that the features of GIMAP1‐deficient T cells that we see in our in vitro culture system mirror what occurs in vivo.

### The survival defect in GIMAP1‐deficient CD4^+^ T cells is not dependent on age

Deletion of *Gimap5* in both rats and mice results in T‐cell lymphopenia, but in mice the strength of this phenotype appears to be age‐dependent. At 4 weeks of age, GIMAP5‐deficient mice show decreased numbers of peripheral CD4^+^ and CD8^+^ T cells 29. This is followed by a further decline in T cells at 6 and 10 weeks of age. Our previous study of GIMAP1 in *Gimap1^f/f^CD2Cre^+^* mice had analysed T‐cell numbers in 10–12 weeks old mice 21, a time point by which any age‐dependent onset of lymphopenia may have already occurred. To determine whether the T‐cell lymphopenia in GIMAP1‐deficient mice also exhibits an age‐dependent profile, we enumerated CD4^+^ T cells in *Gimap1^f/f^CD2Cre^+^* and control *Gimap1^f/f^* mice at 5 and 10 weeks of age. As shown in Fig. 5C, T‐cell numbers in GIMAP1‐deficient mice were profoundly reduced by 5 weeks of age suggesting that the lymphopenia in GIMAP1‐deficient mice is not age‐dependent.

### GIMAP1 is essential for survival of CD4^+^and CD8^+^T cells in vivo

**Figure 6:**
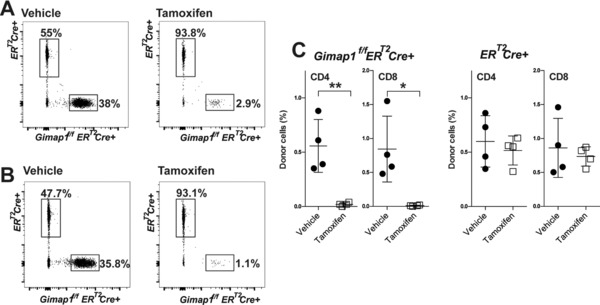
GIMAP1 is required for survival of mature lymphocytes in vivo. (A‐C) Lymph node cells from *Gimap1^f/f^ER^T2^Cre^+^* and control *ER^T2^Cre^+^* were stained with CFSE and Cell Trace^TM^ Violet, respectively. Cell suspensions from each strain were then mixed and co‐injected i.v. into replete C57BL6/SJL‐*PtprcaPepcb*/BoyJ (CD45.1^+^) mice. Recipient mice were injected (i.p. or s.c.) with tamoxifen or vehicle control on days 1 and 2 and the number of remaining transferred cells determined 13 days post cell‐transfer. (A, B) Representative flow cytometry plots showing the percentage of transferred (A) CD4^+^ and (B) CD8^+^ cells from *Gimap1^f/f^ER^T2^Cre^+^* or *ER^T2^Cre^+^* mice following vehicle or tamoxifen treatment. Data shown are from a single experiment representative of three experiments performed. (C) Each symbol represents the percentage of adoptively transferred *Gimap1^f/f^ER^T2^Cre^+^* or *ER^T2^Cre^+^*CD4^+^and CD8^+^cells remaining after vehicle (●) or tamoxifen (□) treatment for individual mice, with the mean ± S.D. shown. Each panel is of two pooled experiments and is representative of three independent experiments. *p < 0.05, **p < 0.005 (unpaired 2‐tailed Student's *t*‐test).

To determine if GIMAP1 is also required continuously for T‐cell survival in vivo, we adoptively transferred mature, peripheral CD4^+^and CD8^+^ T cells from *Gimap1^f/f^ER^T2^Cre^+^* and *ER^T2^Cre^+^* mice into replete, CD45.1‐expressing congenic hosts, which were then treated with tamoxifen to ablate *Gimap1* in the *Gimap1^fl^*‐bearing donor cells. Both *Gimap1^f/f^ER^T2^Cre^+^* and *ER^T2^Cre^+^* mice were on a C57BL/6 background and expressed the CD45.2 allotypic variant. In order to easily differentiate cells from the two strains, *Gimap1^f/f^ER^T2^Cre^+^* and *ER^T2^Cre^+^*lymph node cells were first stained with CFSE and CellTrace^TM^ Violet (CTV), respectively, and then co‐transferred into recipient animals. The recipients were administered tamoxifen on two consecutive days and their spleens harvested 13 days following the adoptive transfer. Splenocytes were stained for CD4, CD8, and CD45.2, and the percentage of donor cells from each strain determined for each recipient animal. As shown in Fig. 6A–C, tamoxifen treatment resulted in a near‐total loss of *Gimap1^f/f^ER^T2^Cre^+^*CD4^+^ and CD8^+^ T cells but had little effect on *ER^T2^Cre^+^* T cells. These results show that CD4^+^and CD8^+^ T cells require continuous intrinsic GIMAP1 expression for their maintenance within the periphery.

## Discussion

The critical importance of the GIMAP family GTPases in the functioning of the immune system is reflected in their association with human diseases. *Gimap* gene expression is silenced or lost in anaplastic T‐cell lymphomas 30, and increased in certain types of leukemia^31^. In addition, GIMAP expression is reduced in regulatory T cells of patients with type I diabetes 32, and a single nucleotide polymorphism in the polyadenylation signal of human *Gimap5* is associated with IA‐2 autoantibody formation in patients with type I diabetes and predisposes individuals to systemic lupus erythematosus 33, 34. Similarly, gene association studies have implicated GIMAPs in other autoimmune diseases such as Behçet's disease 35 and for asthma and allergy sensitization 36. Yet, despite these associations, we still know relatively little about GIMAPs and how they function.

The majority of work in the GIMAP field has focused on GIMAP5 37, 38, 39, 40. The structural similarities between GIMAP5 and GIMAP1 sparked our initial interest in GIMAP1. In previous work, we used conditional knockout technology to specifically delete *Gimap1* from lymphocytes and found a striking similarity in the lymphopenia observed in our *Gimap1*‐deficient T cells with that seen in the BioBreeding rat strain 22. All previous GIMAP knockout models have relied on germline gene mutation or ablation 8, 9, 10, 15, 16, 17. However, mice bearing a homozygous germline knockout of *Gimap1*, although born in normal numbers, rarely survive more than 10 days postnatally (not shown). A further disadvantage to this method of gene ablation is that it potentially confounds the cell‐intrinsic effects of the knock‐out with cell‐extrinsic phenotypes. Our strategy of developing *Gimap1^f/f^* mice has enabled us to examine the cell‐intrinsic effects of the *Gimap1* lesion by controlling where and when the gene is ablated. For the present study, we have generated a tamoxifen‐inducible *Gimap1*knockout model that allows temporal control of gene ablation. A unique advantage gained from using the *Gimap1^f/f^ER^T2^Cre* model is that it allowed us to assay protein turnover and the proficiency of gene ablation. By culturing *Gimap1^f/f^ER^T2^Cre* CD4^+^ T cells in vitro, we found that a low dose of 4‐OHT was sufficient to ablate the floxed *Gimap1* DNA fragment rapidly and completely. Surprisingly, GIMAP1 protein turnover took 6–7 days. In this system, loss of GIMAP1 resulted in cell death. We confirmed this finding in vivo by adoptively transferring *Gimap1^f/f^ER^T2^Cre* or *ER^T2^Cre* lymphocytes into congenic recipient animals and administering tamoxifen. This allowed us to monitor the effect of GIMAP1 deletion on CD4^+^ and CD8^+^ T cells and extended our finding that GIMAP1 maintains the survival of CD4^+^ T cells in vitro. The loss of GIMAP1 similarly compromised the survival of *Gimap1^f/f^ER^T2^Cre* CD8^+^ T cells, both in vivo and in vitro. Thus, mature T cells require continuous GIMAP1 protein expression for their survival. By ablating GIMAP1 protein during the expansion phase of T‐cell activation we also showed that GIMAP1 is critical for the survival of activated T cells. Previous work with GIMAP5‐deficient T cells had shown that antigenic stimulation could rescue GIMAP5‐deficient T cells from death 12, 24, 25. Thus, the cells remaining in the periphery of GIMAP5‐deficient rodents are mainly activated lymphocytes 25, 41, 42. These activated (including potentially autoreactive) cells have a survival advantage, which allows their proliferation in the lymphopenic environment and can mediate autoimmune diseases evident in strains of GIMAP5‐deficient rodents 9, 43. In contrast, we find that GIMAP1‐deficient T cells cannot be rescued from cell death by TCR activation (data not shown) and that activated T cells are still susceptible to death induced by absence of GIMAP1. This finding may explain the lack of autoimmunity observed in the *Gimap1^f/f^CD2Cre^+^* strain of mice and suggests that, despite their structural similarities, GIMAP1 and GIMAP5 perform discrete functions in T cells. In recent work we have looked at the effect of GIMAP1 deletion in B cells and also find that both resting and activated B cells depend on GIMAP1 for their survival 44. Interestingly, attrition in the T‐cell compartments of GIMAP5‐deficient mice is aggravated as the animals age 29. This is not apparent in *Gimap1^f/f^CD2Cre* animals and again suggests that GIMAP1 and GIMAP5 have different specific roles in regulating lymphocyte survival.

Elevated levels of phosphatidylserine exposure and caspase‐3 activity in GIMAP1‐deficient CD4^+^ T lymphocytes initially suggested that these cells were dying by apoptosis. However, the apoptotic pathway by which cells were dying was unclear. The reduction of GIMAP1 protein in cultured CD4^+^ T cells led to mitochondrial depolarization followed by caspase‐8 activation, which suggested that both the intrinsic and extrinsic apoptotic pathways were engaged 26. Ordinarily, the mitochondrial outer membrane is permeabilized by Bax and Bak as part of the intrinsic apoptotic pathway, in response to perturbations in levels of pro‐ and anti‐apoptotic Bcl‐2 family members such as Bcl‐2, Mcl‐1, Bim and PUMA 46. This releases cytochrome c, which forms an integral part of the apoptosome, the caspase‐9 recruitment and activating platform. GIMAPs 3, 4, and 5 have been shown to interact with members of the Bcl‐2 family 14, 45. GIMAP4 co‐immunoprecipitates with Bax^14^. GIMAP5 has been reported to stabilize the interaction between HSC70 and either Mcl‐1 or Bcl‐2 in B cell progenitors, without which the Bcl‐2 family members are rapidly degraded 45. Both GIMAPs 3 and 5 also directly bind Bcl‐x_L_, Bax, Bak, Bad and Bim 14. However, the *Gimap1* lesion did not appear to affect expression of members of this family in CD4^+^ T cells and we saw no activation of caspase‐9. Apoptosis in GIMAP5‐deficient T cells is also not prevented by caspase‐9 inhibitors but is prevented by inhibitors for caspase‐3 and caspase‐8 47. Together, these data suggest that the extrinsic apoptosis pathway is activated in GIMAP1 or GIMAP5‐deficient cells and that the intrinsic pathway remains inert.

In agreement with this finding, the few CD4^+^and CD8^+^ T cells remaining in *Gimap1^f/f^CD2Cre^+^* mice demonstrated elevated levels of activated caspase‐8 and annexin V, confirming our in vitro findings. The extrinsic apoptotic pathway is triggered by healthy cells instructing damaged or infected cells to die and may also be a back‐up mechanism for cells that have escaped deletion by the intrinsic pathway 48. We postulate that the loss of GIMAP1 results in mitochondrial damage, which triggers the activation of caspase‐8. However, the initial injury (mitochondrial damage) is one from which the cells cannot recover. Therefore, caspase‐8 inhibition can prevent the induction of apoptosis (as measured by annexin V binding), but cannot prevent the inevitable cell death that results from mitochondrial damage. It is unclear how GIMAP1 deficiency causes mitochondrial damage. GIMAP1 is localized to the trans‐Golgi network 49. A GIMAP1 lesion may destabilize the Golgi, resulting in the breakdown of endocytic traffic to and from this compartment and leading to the premature release of cathepsins and lysosomal hydrolases into the cytosol (cathepsin D, released from lysosomes during apoptosis induction, has been shown to activate caspase‐8 50.) These events could diminish the efficiency of degradative (lysosomal) pathways including mitophagy, and so lead to the accumulation of damaged mitochondria. It is worth noting findings on the IFN‐γ‐inducible regulatory immunity‐related GTPase (IRG) Irgm1, which is mainly localized at the lysosomal and Golgi membranes. Interestingly, deletion of Irgm1 results in a lympho‐myeloid collapse in response to infection and in vitro is associated with reduced lysosomal acidity and a disruption of autophagic flux 51, 52.

Another puzzling feature of the GIMAP1‐deficiency phenotype has been why the T cells mainly succumb to death in the periphery. GIMAP1 is highly expressed during all stages of T‐cell development in the thymus yet only the most mature SP thymocytes show a survival defect 22, 49. Furthermore, whilst *Gimap1^f/f^CD2Cre^+^* mice have around 30% of mature SP thymocytes, they have <5% of peripheral CD4^+^ T cells 22. This suggests that there is a greater requirement for GIMAP1 in the periphery. It is possibly that the mechanism through which GIMAP1 acts is only active in mature lymphocytes or that there is a factor in the periphery that is dependent upon GIMAP1 function to aid long term survival of mature CD4^+^ T cells. Recent work has suggested that loss of mitochondrial electron chain transport function has a greater effect on peripheral T cells than on thymocytes 53. Similar to GIMAP1 deletion, conditional ablation of apoptosis inducing factor (AIF) results in normal thymocyte development but greatly reduced numbers of peripheral T cells, with CD8^+^ T cells being more affected than CD4^+^ T cells 53. It is tempting to speculate that the differential susceptibility of peripheral T cells to the absence of GIMAP1 is similarly related to the requirements of maintaining mitochondrial function and health.

## Materials and methods

### Animals

Mice were bred and maintained in specific pathogen‐free conditions at The Babraham Institute. Husbandry and experimentation complied with existing United Kingdom Home Office and European Union legislation, as well as local standards, as approved by the Babraham Institute Animal Welfare and Ethical Review Body. *Gimap1^f/f^* mice (described previously 22), bearing a floxed *Gimap1* allele, were crossed with *ER^T2^Cre^+^* mice (obtained from Thomas Ludwig; 54) to generate *Gimap1^f/f^ER^T2^Cre^+^* mice, enabling conditional ablation of *Gimap1* upon administration of tamoxifen or its derivative, 4‐OHT.

In adoptive‐transfer experiments, lymph node cells from *Gimap1^f/f^ER^T2^Cre^+^* and *ER^T2^Cre^+^* mice were stained with CFDA‐SE (Life Technologies) and CTV (Life Technologies), respectively, and then mixed in a 1:2 ratio (*Gimap1^f/f^ER^T2^Cre^+^:ER^T2^Cre^+^*) prior to i.v. injection of 5 × 10^6^ cells/mouse into B6.SJL‐*Ptprca Pepcb/BoyJ mice*. Mice were treated with 200 μg tamoxifen/g bodyweight in sunflower oil, or vehicle control, i.p. or s.c. on days 1 and 2 following adoptive cell transfer. On day 13 after cell transfer, mice were killed and the numbers of transferred cells present in peripheral blood and spleen were determined.

### PCR and western blot analysis

The presence of the *Gimap1^f/f^* allele and deletion of *Gimap1* were determined by PCR as previously described 22. Ablation of GIMAP1 protein was determined by western blot as previously described 22.

### CD4^+^ T‐cell enrichment and culture

CD4^+^ lymphocytes were purified from spleens and lymph nodes of *Gimap1^f/f^ER^T2^Cre* ^+^and *ER^T2^Cre* ^+^mice by negative selection. In brief, erythrocytes were lysed prior to incubation of cells with biotinylated anti‐CD19, anti‐IgD, anti‐CD8, anti‐CD25, anti‐NK1.1, anti‐γδTCR, anti‐Gr1, and anti‐CD11b. Cells were washed and then incubated with anti‐biotin MACS beads prior to depletion of bead‐coated cells by AutoMACS (MiltenyiBiotec). Cells were then cultured at 1–2 × 10^6^ cells/ml in 10% FCS IMDM (culture medium) with 5 ng/mL IL‐7 (Peprotech) with vehicle (DMSO) or 50 nM 4‐OHT. After 48 h, cells were washed and resuspended at 1–2 × 10^6^ cells/mL in culture medium with 5ng/mL IL‐7 for a further 2–5 days. Q‐VD‐OPh hydrate (QVD; Calbiochem) and Z‐Ile‐Glu(O‐ME)‐Thr‐Asp(O‐Me) fluoromethyl ketone (IETD; Sigma Aldrich) were added to give final concentrations of 10 μg/mL and 100 μg/mL, respectively, on days 6 and 8 of culture. To determine proliferation, cells were first stained with CFSE according to the manufacturer's instructions. Cells were then cultured at a concentration of 1–2 × 10^6^ cells/mL, in anti‐CD3 (eBioscience)‐coated 24‐ or 96‐well plates with anti‐CD28 (eBioscience; 100ng/mL) plus IL‐2 (RnD Systems; 10 ng/mL) for 24–120 h prior to analysis by flow cytometry. To determine oxygen consumption rate, a Seahorse Extracellular Flux (XF) 24 Analyser (Seahorse Bioscience) was used, as previously described 16.

### Flow cytometry

Single cell suspensions then stained with fluorochrome‐conjugated anti‐CD4, anti‐CD8 and anti‐CD25 mAbs (BD Biosciences and eBioscience). For detection of activated caspases, CaspGLOW Fluorescein Active Caspase‐3, Caspase‐8 or Caspase‐9 kits (Biovision) were used according to the manufacturer's instructions. Cells were stained for annexin V and DAPI by incubating with annexin V conjugates (BD Biosciences) and DAPI (Sigma) in annexin V binding buffer (BD Biosciences). To determine mitochondrial health, cells were incubated with JC‐1 (Molecular Probes) for 30 min at 37°C prior to analysis. Cells were counted using either a CASY^®^ cell counter and analyzer system (Roche Innovatis AG) or Flow Count beads (Sigma) during flow cytometric analysis. Flow cytometry was performed using a BD LSRII or Fortessa cell analyser (BD), and data were analyzed using Flowjo analytic software.

## Conflict of interest

The authors declare no financial or commercial conflict of interest.

## Supporting information

As a service to our authors and readers, this journal provides supporting information supplied by the authors. Such materials are peer reviewed and may be re‐organized for online delivery, but are not copy‐edited or typeset. Technical support issues arising from supporting information (other than missing files) should be addressed to the authors.

Supplementary Figure 1.

Supplementary Figure 2.
