# Supplementary figures and images for: Survival of mature T cells in the periphery is intrinsically dependent on GIMAP1 in mice
Source: Eur J Immunol. 2016 Nov 25;47(1):84–93. doi: 10.1002/eji.201646599 (PMC5244661; doi:10.1002/eji.201646599)

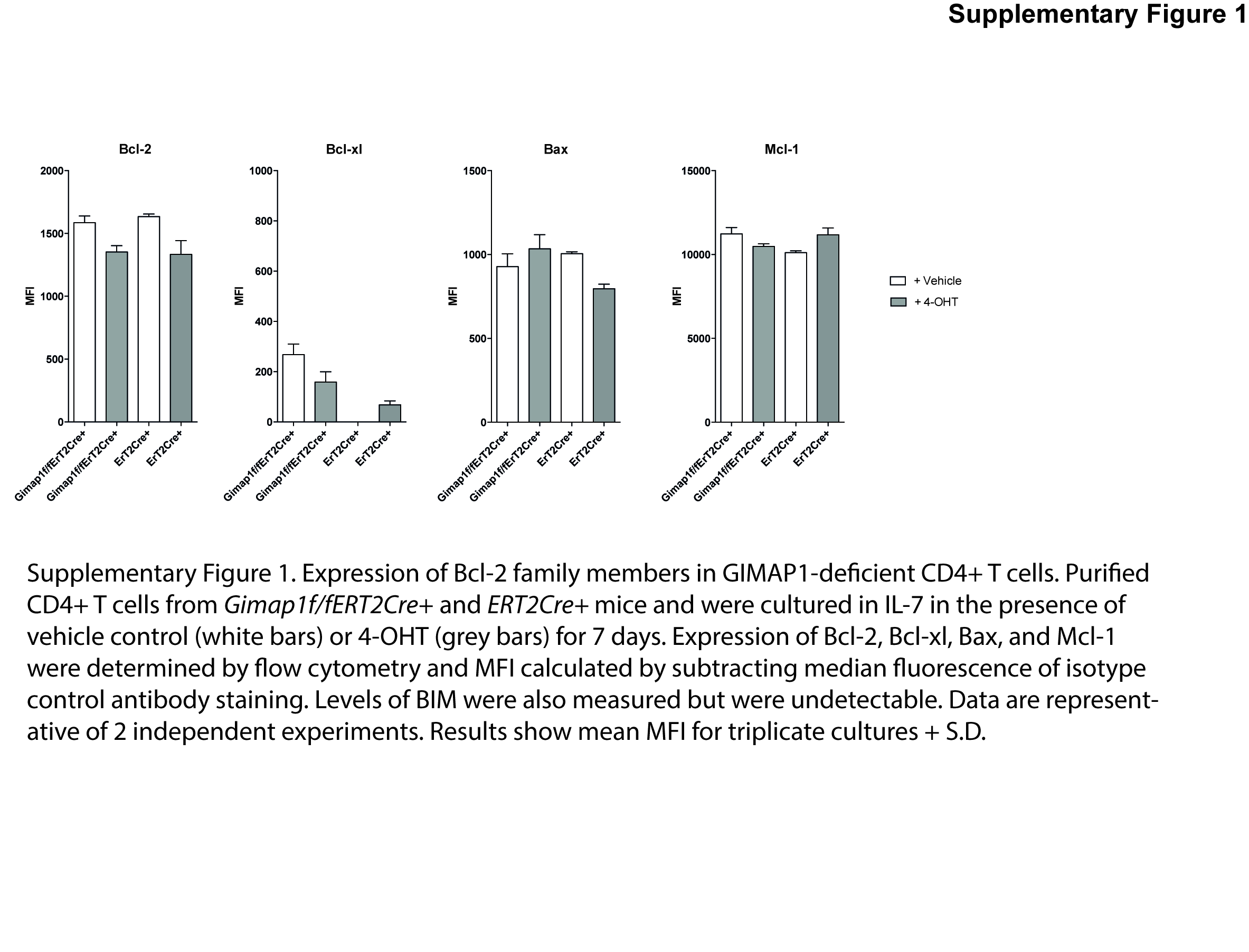

Supplement: Supplementary file 1 — Supplementary Figure 1. [file EJI-47-84-s001.tif]
