# Supplementary material for: Survival of mature T cells in the periphery is intrinsically dependent on GIMAP1 in mice
Source: Eur J Immunol. 2016 Nov 25;47(1):84–93. doi: 10.1002/eji.201646599 (PMC5244661; doi:10.1002/eji.201646599)
Supplement: Supplementary file 2 — Supplementary Figure 2. [file EJI-47-84-s002.pdf]

Peer review correspondence

Survival of mature T cells in the periphery is intrinsically dependent on GIMAP1 in mice

Preeta Datta, Louise M.C. Webb, Inxhina Avdo, John Pascall and Geoffrey W. Butcher

Corresponding author: Geoffrey W. Butcher, Laboratory of Lymphocyte Signalling and Development, The Babraham Institute, Cambridge, United Kingdom

---

Review Timeline:	Submission date:	11 July 2016
	First editorial decision:	22 August 2016
	Revision received:	8 September 2016
	Accepted:	26 October 2016

Handling Executive Committee member: Prof. Shimon Sakaguchi

Please note that the correspondence below does not include the standard editorial instructions regarding preparation and submission of revised manuscripts, only the scientific revisions requested and addressed.

First Editorial Decision – 22 August 2016

Dear Dr. Webb,

My apologies for the slight delay in processing the peer review of your manuscript ID eji.201646599 entitled "Mature T cells are intrinisically dependent upon GIMAP1 for their survival in the periphery", which you submitted to the European Journal of Immunology. There was a delay in receiving one of the reports; nevertheless all opinions have been received and the comments of the referees are included at the bottom of this letter.

Although the referees have recommended publication, some revisions to your manuscript have been requested. Therefore, I invite you to respond to the comments of the referees and revise your manuscript accordingly.

You should also pay close attention to the editorial comments included below. *In particular, please edit your figure legends to follow Journal standards as outlined in the editorial comments. Failure to do this will result in delays in the re-review process.*

If the revision of the paper is expected to take more than three months, please inform the editorial office. Revisions taking longer than six months may be assessed by new referee(s) to ensure the relevance and timeliness of the data.

Once again, thank you for submitting your manuscript to European Journal of Immunology. We look forward to receiving your revision.

Yours sincerely,
Karen Chu

on behalf of Prof. Shimon Sakaguchi

Dr. Karen Chu
Editorial Office
European Journal of Immunology
e-mail: ejied@wiley.com
www.eji-journal.eu

****

Reviewer: 1
Comments to the Author
This manuscript by Butcher and his colleagues nicely show that Gimap1 plays an essential role in keeping the survival of naïve T cells (both CD4 and CD8 T cells) in the periphery. Their inducible Cre-mediated deletion of Gimap1 specifically in T cells and careful control of the measurements convincingly support the results and conclusions. The data on caspase 8 and mitochondrial potential are interesting. The results are important and should warrant a decent publication. I only have a few minor comments as follows.

1. The data on isolated naïve T cells are important. It would be nice to additionally address the role of GIMAP1 in the maintenance of memory-like T cells isolated in parallel from normal mice.

2. Page 9, line 8: “As shown in 9A & C” may mean “As shown in Figures 4A & C”?

Reviewer: 2
Comments to the Author
In this study the authors look at the mechanisms of GIMAP1 in lymphocyte function and survival and use a novel tamoxifen-inducible mouse model in order to distinguish the effects of GIMAP1 knockdown in

European Journal of Immunology

mature vs developing lymphocytes. The authors conclude that GIMAP1 plays an essential role in mature peripheral lymphocytes and is intrinsically required for their survival. Although the study is in general well designed and performed there are still few comments related to experimental setup and presentation of the results.

Comments:
Although the authors have developed a novel inducible GIMAP1 KO mouse model with clear advantages over the conditional GIMAP KO, some of the key findings (ie in vivo characterization of lymphocyte apoptosis and caspase-8) are still done in the constitutive model. These experiments should be done also in the tamoxifen-inducible GIMAP1 KO vs controls.

Also, the experiments on mitochondrial function (shown using the ex vivo model) should be shown in the tamoxifen-inducible GIMAP1 KO model in vivo.

There is no data given on the number of performed experiments in Fig2 and respective legend. Also, the legend does not state whether SEM or SD has been used. This info should be included in the figure legend.

In some figures the data are presented as mean with SD and in some as mean with SEM. Unless there is a clear purpose for this approach the authors should choose either SEM of SD for all data.

The last sentence in Figure legend 5 states that ERT2Cre model has been used whereas the rest of the text indicates CD2Cre.

Page 9, para 2 incorrectly refers to Fig 9 A&B. This should be corrected.

First revision – authors' response – 8 September 2016

Reviewer: 1

Comments to the Author
This manuscript by Butcher and his colleagues nicely show that Gimap1 plays an essential role in keeping the survival of naïve T cells (both CD4 and CD8 T cells) in the periphery. Their inducible Cre-mediated deletion of Gimap1 specifically in T cells and careful control of the measurements convincingly support the results and conclusions. The data on caspase 8 and mitochondrial potential are interesting. The results are important and should warrant a decent publication. I only have a few minor comments as follows.

European Journal of Immunology

1. The data on isolated naïve T cells are important. It would be nice to additionally address the role of GIMAP1 in the maintenance of memory-like T cells isolated in parallel from normal mice.

This is a pertinent question that we would like to address. Ideally, we would delete GIMAP1 from memory T cells using a model antigen system both in vivo and in vitro. This requires generation of TCR transgenic mice on the GIMAP1f/fERT2Cre+ mice. We feel that the time involved and the amount of additional data to adequately address this question are beyond the scope of this manuscript.

2. Page 9, line 8: “As shown in 9A & C” may mean “As shown in Figures 4A & C”?

Yes, this has been amended.

We thank the reviewer for their comments.

Reviewer: 2

Comments to the Author

In this study the authors look at the mechanisms of GIMAP1 in lymphocyte function and survival and use a novel tamoxifen-inducible mouse model in order to distinguish the effects of GIMAP1 knockdown in mature vs developing lymphocytes. The authors conclude that GIMAP1 plays an essential role in mature peripheral lymphocytes and is intrinsically required for their survival. Although the study is in general well designed and performed there are still few comments related to experimental setup and presentation of the results.

Comments:

Although the authors have developed a novel inducible GIMAP1 KO mouse model with clear advantages over the conditional GIMAP KO, some of the key findings (ie in vivo characterization of lymphocyte apoptosis and caspase-8) are still done in the constitutive model. These experiments should be done also in the tamoxifen-inducible GIMAP1 KO vs controls.

These are attractive ideas but beyond the scope of the present study. Our main findings (that GIMAP1 deletion results in (apoptosis and increased levels of active Caspase 8) have been shown in vitro using inducible deletion of GIMAP1 and then confirmed in vivo using conditional knockout of GIMAP1. To repeat these experiments in tamoxifen-inducible GIMAP1KO vs controls in vivo would only confirm these findings and would not advance our understanding of GIMAP1 function. Experiments along these lines would require substantial preliminary tests to determine the detailed in vivo kinetics (and variance) of changes accompanying in vivo tamoxifen injection of inducible GIMAP1 KO mice and whether these were compatible with meaningful data collection. Another unpredictable confounding factor could be the early in vivo detection and clearance of cells destined to die.

Also, the experiments on mitochondrial function (shown using the ex vivo model) should be shown in the tamoxifen-inducible GIMAP1 KO model in vivo.

As above, this is an attractive idea but experiments along these lines would require substantial preliminary tests to determine the detailed in vivo kinetics (and variance) of changes accompanying in vivo tamoxifen injection of inducible GIMAP1 KO mice and whether these were compatible with meaningful data collection. Another unpredictable confounding factor could be the early in vivo detection and clearance of cells destined to die.

There is no data given on the number of performed experiments in Fig2 and respective legend. Also, the legend does not state whether SEM or SD has been used. This info should be included in the figure legend.

This has now been amended.

In some figures the data are presented as mean with SD and in some as mean with SEM. Unless there is a clear purpose for this approach the authors should choose either SEM of SD for all data.

We have used standard deviation when the samples shown are from different mice, i.e. biological replicates as standard deviation is a measure of the amount of variation within a population and informs us about the spread of the population. When we have shown technical replicates the standard error of the mean is shown. The standard error of the mean shows the uncertainty in the mean and its dependency on the sample size, n (s.e.m. = s.d./√n) and is more appropriate for technical replicates than standard deviation. (Ref http://www.nature.com/nmeth/journal/v10/n10/full/nmeth.2659.html).

The last sentence in Figure legend 5 states that ERT2Cre model has been used whereas the rest of the text indicates CD2Cre.

This is correct, and the manuscript has now been amended.

Page 9, para 2 incorrectly refers to Fig 9 A&B. This should be corrected.

This has now been amended.

We thank the reviewer for their comments.

Second Editorial Decision – 7 October 2016

Dear Dr. Webb, Dr. Butcher,

It is a pleasure to provisionally accept your manuscript entitled "Survival of mature T cells in the periphery is intrinisically dependent upon GIMAP1 in mice" for publication in the European Journal of Immunology. For final acceptance, please follow the instructions below and return the requested items as soon as possible as we cannot process your manuscript further until all items listed below are dealt with.

Please note that EJI articles are now published online a few days after final acceptance (see Accepted Articles: http://onlinelibrary.wiley.com/journal/10.1002/(ISSN)1521-4141/accepted). The files used for the Accepted Articles are the final files and information supplied by you in Manuscript Central. You should

therefore check that all the information (including author names) is correct as changes will NOT be permitted until the proofs stage.

We look forward to hearing from you and thank you for submitting your manuscript to the European Journal of Immunology.

Yours sincerely,
Karen Chu

on behalf of Prof. Shimon Sakaguchi

Dr. Karen Chu
Editorial Office
European Journal of Immunology
e-mail: ejied@wiley.com
www.eji-journal.eu
